# Supplementary material for: A fault-intrusion-tolerant system and deadline-aware algorithm for scheduling scientific workflow in the cloud
Source: PeerJ Comput Sci. 2021 Nov 2;7:e747. doi: 10.7717/peerj-cs.747 (PMC8576554; doi:10.7717/peerj-cs.747)
Supplement: Supplemental Information 6 [file peerj-cs-07-747-s006.zip › Myproject1.1/docs/org/cloudbus/cloudsim/power/models/PowerModelSpecPowerIbmX3250XeonX3470.html]

PowerModelSpecPowerIbmX3250XeonX3470


JavaScript is disabled on your browser.


- Overview
- Package
- Class
- Use
- Tree
- Deprecated
- Index
- Help

- Prev Class
- Next Class

- Frames
- No Frames

- All Classes

- Summary:
- Nested |
- Field |
- Constr |
- Method

- Detail:
- Field |
- Constr |
- Method


org.cloudbus.cloudsim.power.models

## Class PowerModelSpecPowerIbmX3250XeonX3470

- java.lang.Object
- - org.cloudbus.cloudsim.power.models.PowerModelSpecPower
  - - org.cloudbus.cloudsim.power.models.PowerModelSpecPowerIbmX3250XeonX3470

- All Implemented Interfaces:
  :   PowerModel

  ---

    

  ```
  public class PowerModelSpecPowerIbmX3250XeonX3470
  extends PowerModelSpecPower
  ```

  The power model of an IBM server x3250 (1 x [Xeon X3470 2933 MHz, 4 cores], 8GB).
  http://www.spec.org/power\_ssj2008/results/res2009q4/power\_ssj2008-20091104-00213.html
  If you are using any algorithms, policies or workload included in the power package, please cite
  the following paper:
  Anton Beloglazov, and Rajkumar Buyya, "Optimal Online Deterministic Algorithms and Adaptive
  Heuristics for Energy and Performance Efficient Dynamic Consolidation of Virtual Machines in
  Cloud Data Centers", Concurrency and Computation: Practice and Experience (CCPE), Volume 24,
  Issue 13, Pages: 1397-1420, John Wiley & Sons, Ltd, New York, USA, 2012

  Since:
  :   CloudSim Toolkit 3.0

- - ### Constructor Summary

    Constructors

    Constructor and Description

    `PowerModelSpecPowerIbmX3250XeonX3470()`
  - ### Method Summary

    Methods

    | Modifier and Type | Method and Description |
    | --- | --- |
    | `protected double` | `getPowerData(int index)` Gets the power data. |

    - ### Methods inherited from class org.cloudbus.cloudsim.power.models.PowerModelSpecPower

      `getPower`
    - ### Methods inherited from class java.lang.Object

      `clone, equals, finalize, getClass, hashCode, notify, notifyAll, toString, wait, wait, wait`

- - ### Constructor Detail


    - #### PowerModelSpecPowerIbmX3250XeonX3470

      ```
      public PowerModelSpecPowerIbmX3250XeonX3470()
      ```
  - ### Method Detail


    - #### getPowerData

      ```
      protected double getPowerData(int index)
      ```

      **Description copied from class: `PowerModelSpecPower`**

      Gets the power data.

      **Specified by:**
      :   `getPowerData` in class `PowerModelSpecPower`

      Parameters:
      :   `index` - the index

      Returns:
      :   the power data


- Overview
- Package
- Class
- Use
- Tree
- Deprecated
- Index
- Help

- Prev Class
- Next Class

- Frames
- No Frames

- All Classes

- Summary:
- Nested |
- Field |
- Constr |
- Method

- Detail:
- Field |
- Constr |
- Method
